# Training in Language Switching Facilitates Bilinguals’ Monitoring and Inhibitory Control

**DOI:** 10.3389/fpsyg.2019.01839

**Published:** 2019-08-13

**Authors:** Cong Liu, Chin-Lung Yang, Lu Jiao, John W. Schwieter, Xun Sun, Ruiming Wang

**Affiliations:** ^1^Guangdong Provincial Key Laboratory of Mental Health and Cognitive Science, Center for Studies of Psychological Application, School of Psychology, South China Normal University, Guangzhou, China; ^2^Department of Psychology, Qingdao University, Qingdao, China; ^3^Department of Linguistics and Modern Languages, Chinese University of Hong Kong, Hong Kong, China; ^4^Language Acquisition, Multilingualism, and Cognition Laboratory, Wilfrid Laurier University, Waterloo, ON, Canada

**Keywords:** bilingualism, language switching training, cognitive control, monitoring, inhibitory control, positive psychology

## Abstract

In the present study, we use a training design in two experiments to examine whether bilingual language switching facilitates two components of cognitive control, namely monitoring and inhibitory control. The results of Experiment 1 showed that training in language switching reduced mixing costs and the anti-saccade effect among bilinguals. In Experiment 2, the findings revealed a greater decrease of mixing costs and a smaller decrease of the anti-saccade effect from pre- to post-training for the language switching training group compared to the second language training group. Overall, the results suggest that extensive exercise in monitoring and inhibitory control in an experimental setting may enhance the corresponding components of cognitive control. We discuss these findings in the context of the relationship between bilingual language control and executive control.

## Introduction

Bilinguals constantly switch between the languages they speak in their multilingual societies, according to the needs of the interactional contexts. Given that previous studies have indicated that both languages are activated in parallel during bilingual speech production (Liu et al., 2019b; for a review, see Declerck and Philipp, 2015), and that during language switching, when bilinguals speak in the intended language, they utilize language control mechanisms to inhibit the interference from the other language. The extent to which this bilingual language control (BLC) overlaps with control processes implemented in non-linguistic processing has been debated for years (e.g., Declerck et al., 2017; Segal et al., 2019; for a review, see Calabria et al., 2019).

Some behavioral correlational studies have shown a clear link between BLC and domain-general executive control (EC) as revealed by a correlation between performances on the two control tasks (Prior and Gollan, 2013; Declerck et al., 2017; Timmer et al., 2018), whereas others have not (Calabria et al., 2015; Branzi et al., 2016). Subsequent neuroimaging studies further have revealed the similarities between the underlying brain networks of BLC and EC (De Baene et al., 2015; Weissberger et al., 2015), but only in a limited number of studies. More recently, some studies started to investigate this relationship by assessing the effect of short language switching training on task switching (Prior and Gollan, 2013; Timmer et al., 2019). The evidence regarding cross-talk that comes from training studies is based on the idea that if the two controls share cognitive processes, then training in BLC during language switching would transfer to EC. The findings from Timmer et al. suggested that short-term language switching training indeed transfers to the non-linguistic domain for certain sub-mechanisms (i.e., switch cost) but not for others (mixing cost). However, different results were reported in Prior and Gollan’s study in which there was no transfer effect from language switching training to EC performance (neither for switch cost nor mixing cost). Thus, it is still an open question as to whether BLC overlaps with EC.

The present study addresses this issue by examining the effects of short-term language switching training on monitoring and inhibitory control among Chinese (L1)-English (L2) bilinguals. In the next section, we provide a background of some of the work that has recently examined monitoring and inhibitory control with regard to BLC and EC. We then describe the two experiments in the present study including the participants, design, procedures, and experimental tasks. Finally, we discuss the findings, implications, and suggestions for future work.

## Background

### Conflict Monitoring and Inhibitory Control

Conflict monitoring (monitoring in short below; Jylkkä et al., 2018; Struys et al., 2019) and inhibitory control (Babcock and Vallesi, 2015; Declerck and Philipp, 2015) are two main processing systems used in language switching (Hartanto and Yang, 2019). Specifically, bilinguals monitor the conflict (i.e., cross-language interference) in the communicative environment for changes that trigger a language switch, and then instigate the language control process to inhibit cross-language interference. Given that monitoring and inhibitory control are essential to language switching, we focus on these two processing components in the present study.

Monitoring is the ability to detect a potential conflictive situation and signal that the situation demands a specific action (Costa et al., 2008). The global response time (RT) and mixing costs within a switch task are two different indexes that are used to measure monitoring. While global RT refers to the overall faster responses (Hartanto and Yang, 2019), mixing costs refers to the performance difference between single-language blocks which do not involve monitoring and repetition trials of mixed-language blocks which place high demands on monitoring (Braver et al., 2003; Prior and MacWhinney, 2010; Jylkkä et al., 2018). Ma et al. (2016) indicated that mixing costs also reflect proactive control during word production. In the current study, we used the latter index as it has been argued to be a purer measure of monitoring than global RT (Paap and Greenberg, 2013).

Inhibitory control is the ability to control one’s attention, behavior, or internal predisposition for executing appropriate responses (Clark, 1996; Bunge et al., 2002). There are various indexes from different tasks to measure inhibitory control. For example, the flanker effect in the flanker task, the Simon effect in the Simon task, or the anti-saccade effect in the anti-saccade task (Paap and Sawi, 2014; Jiao et al., 2019). Here, we chose the anti-saccade task because it is a more difficult task (Hamilton and Martin, 2005) that could avoid ceiling effects. The anti-saccade effect refers to the performance difference between anti-saccade blocks which place a high demand on inhibitory control and control blocks which do not involve inhibitory control (Paap and Greenberg, 2013).

According to the adaptive control hypothesis (Green and Abutalebi, 2013), control processes adapt to the demands imposed on them by context. In this way, certain components of EC would show a bilingual facilitative effect when the context imposes demands on it. In the present study, we examine whether the specific exercise in monitoring and inhibitory control during language switching transfers to the corresponding parts of domain-general cognitive control.

### Coss-Talk Between BLC and EC

Previous studies have introduced two theoretical accounts to explain the relationship between BLC and the two components of EC (i.e., monitoring and inhibitory control). These explanations include the bilingual executive processing advantage hypothesis (Hilchey and Klein, 2011) and the inhibitory control model (Green, 1998). The former postulates that bilinguals are better at global conflict-monitoring than monolinguals because of their constant practice with coordination of two competitive languages which should be beneficial for the monitoring aspects of cognitive control.^1^ The inhibitory control model (Green, 1998) postulates that because two languages are coactivated automatically and compete with each other in the mind, bilinguals constantly recruit inhibitory control mechanisms to select the intended language while suppressing the irrelevant language. Bilinguals’ routine practice of inhibiting the irrelevant language should be beneficial for the inhibitory control aspects of EC.

In line with the bilingual executive processing advantage hypothesis (Hilchey and Klein, 2011) and the inhibitory control model (Green, 1998), many previous studies have revealed a positive relationship between BLC and monitoring (e.g., Costa et al., 2009) and between BLC and inhibitory control (e.g., Bialystok et al., 2006; Fan et al., 2012). However, other studies have found no such relationship (Prior and MacWhinney, 2010; Paap and Greenberg, 2013). For example, Paap and Greenberg compared bilinguals to monolinguals on 15 indicators of executive processing (EP). The results showed no bilingual facilitative effect on any indicator suggesting no relationship between BLC and EC.

One possible reason for the inconsistent findings regarding the cross-talk between BLC and EC is due to the experimental design. Most studies examining the relationship between bilingualism and cognitive control directly compared the performance of a bilingual group and a monolingual group. Such cross-sectional designs involve larger individual differences and might not reveal the causal relationship between bilingualism and cognitive control. However, a training design may better match the background information of different groups, and benefit the assessment of causal relation between the language experience and cognitive capabilities (Li and Grant, 2015) because it enables the tracking of basic comprehension processes such as word knowledge/processes in the second language (Grant et al., 2015; Yang et al., 2015) and of the effects of linguistic and non-linguistic control, respectively, in the development of cognitive control (Moreno et al., 2011).

For instance, in a training setting Zhang et al. (2015) examined the effects of language switching on the component processes of cognitive control (i.e., proactive control and reactive control; Braver, 2012). Participants were randomly and evenly divided into experimental and control groups. Both groups completed the same cognitive control task (i.e., the AX version of the Continuous Performance Test in Braver and Barch, 2002, and Rosvold et al., 1956) in the pre- and post-training phase whereas only experimental group received a training phase which included a series of language switching training in between the pre- and post-training phases. The results showed that the language switching experience facilitated cognitive control, suggesting a positive relationship between BLC and EC. By comparing the task performance between the pre- and post-training phase and by comparing the experimental group with the control group, this facilitative effect on cognitive control could be more straightforwardly attributable to the effect of language switching training.

## The Present Study

In the present study, we explore the effect of language switching on monitoring and inhibitory control by adopting a training design. We conducted two experiments, both of which included, sequentially, a pre-training phase, a training phase, and a post-training phase. In Experiment 1, both groups of participants were required to complete two cognitive control tasks (i.e., a color-shape switching task and an anti-saccade task) that are useful in assessing the two components processes of EC of interest in pre- and post-training phases. The language switching training group received a series of language switches in the training phase and hence engaged monitor and inhibition processes, whereas the control group received no training. Hence, by comparing the language switching training group’s performance in the pre- and post-training phase, we can assess the potential effect of language switching training on the monitoring (i.e., mixing costs in the color-shape switching task) and inhibitory control (i.e., anti-saccade effects in the anti-saccade task). We hypothesize that if BLC and these two components of EC share some processes, then the training on BLC should lead to benefits in EC. Moreover, because the language switching training group trained specifically on monitoring and inhibition during language switching while the control group did not train on these abilities, we expect a greater decrease of the anti-saccade effect and mixing cost from pre- to post-training for the language switching training group compared to the control group.

In Experiment 2, we further explored which factors contribute to the positive relationship between BLC and EC. Specifically, we aimed to reveal where the facilitative effect on monitoring or inhibitory control derived from: the specific training on monitoring, the specific training on inhibitory control, or both. Two different groups of participants were asked to complete two cognitive control tasks similar to those in the pre- and post-training phase in Experiment 1. In the training phase, one group of participants received a series of language switches engaging monitoring and inhibitory control processes (language switching training group), whereas the other group received training which only engaged inhibitory control processing (L2 training group). If the facilitative effect is only derived from training on monitoring during language switching, there will be a greater decrease of mixing costs and anti-saccade effect from pre- to post-training for the language switching training group compared to the L2 training group. However, if the facilitative effect is only derived from training on inhibitory control during language switching, there will be a greater decrease of mixing costs and anti-saccade effect from pre- to post-training for the L2 training group compared to the language switching training group. Furthermore, if the facilitative effect derived from training on both monitoring and inhibitory control during language switching, there will be a greater decrease of mixing cost and smaller decrease of anti-saccade effect from pre- to post-training for the language switching training group compared to the L2 training group.

## Experiment 1

### Participants

Sixty Chinese-English bilinguals (all non-English majors) at the South China Normal University (SCNU), aged 18–24: *M* = 20.6, *SD* = 1.2, participated in Experiment 1. These individuals were all right-handed with normal or corrected-to-normal vision. All participants were English-as-a-foreign-language (EFL) learners whose mean age of first exposure was 9 years, *SD* = 1.7. Participants were asked to complete a questionnaire about their language background, including the age of L2 acquisition (L2 AoA), L2 proficiency, and the frequency of language switching (see Table 1). The participants rated their L2 proficiency level on a seven-point Likert scale, with seven indicating the highest level of proficiency and one indicating the lowest. They also rated their frequency of language switching on a five-point Likert scale, with five indicating the highest frequency of language switching and one indicating the lowest. All participants were Han Chinese without experience with immigration. Participants were divided, evenly and randomly, into two groups (language switching training vs. control) at first, but there was one participant in the language switching group who did not undergo language switching training between pre- and post-training, which lead to 29 participants in the language switching group and 31 participants in the control group. Additionally, two participants in the language switching group were excluded from data analysis due to test incompletion, eventually leaving 27 for the language switching training group and 31 for the control group. Pair-wise *t*-tests revealed no significant differences between the two groups regarding their language background information (*p* > 0.05). The study was approved by the Human Research Ethics Committee of South China Normal University. All participants provided informed consent before the start of the experiment and were informed of their right to withdraw at any time. Participants received monetary compensation for their participation.

**TABLE 1:** Language background information for participants in Experiment 1.

	**L2 AoA**	**L2 proficiency**	Frequency of language switching^*^
Control group	9.1 ± 1.8	4.5 ± 1.14	2.7 ± 0.4
Switching training group	9.6 ± 1.8	4.9 ± 1.33	2.6 ± 0.4

^*^As measured by a language switching questionnaire (Rodriguez-Fornells et al., 2012).

### Design and Procedure

The experiment involved three phases: (1) a pre-training phase, in which participants of both language switching training and control groups were asked to complete the language questionnaire and then to participate in two non-linguistic cognitive control tasks (i.e., the color-shape switching task and the anti-saccade task which measure monitoring and inhibitory control, respectively); (2) a training phase, in which only the language switching training group received language switching training (linguistic switching-task training) for a total of eight times on four consecutive days, two times per day. It took approximately 30 min for each participant to receive the language-switching training each day; and 3) a post-training phase, held on the day after the training, in which all participants in both groups once again performed the same two non-linguistic cognitive control tasks. The waiting period between pre- and post-training was the same for both the control group and language switching training group.

Two strategies were adopted to minimize potential confounds that may undermine the effects of language-switching training on the non-verbal tasks of cognitive control when comparing the performance differences between the pre-training and the post-training phases and between the language switching training and the non-training (control) groups. First, to mitigate differences in task expectancy between the experimental and control groups, both groups were offered no explicit information regarding the types and sequence of tasks being administered in each test phase (i.e., pre- and post-training). They were merely informed, during recruitment, that they would be taking a set of tests twice. The sequence of the two non-linguistic cognitive control tasks in the pre- and post-training phases was also randomly assigned for each participant. Half of the participants started with color-shape switching task and the other half started with anti-saccade tasks. Furthermore, because it has been shown that goal-imposed tasks, verbal or non-verbal, potentially enhance the abilities associated with the cognitive control for subsequent cognitive processes (Gratton et al., 1992; Ullsperger et al., 2005; Wu and Thierry, 2013), we opted to offer neither linguistic nor non-linguistic tasks for the control group during the training phase.

#### Language Switching Training Task

Only participants in the language switching training group received this training task. We used the cued naming task as the language switching training task because the process of language production has been shown to be highly related to cognitive control abilities (Liu et al., 2015; Sikora et al., 2016; Lu et al., 2017). This task was conducted between the pre-training and post-training phases, and required participants to name a target number (e.g., Arabic numerals 0–9), presented on the computer screen, in their L1 (Mandarin) and L2 (English) according to flag cues (Chinese flag for L1; American flag for L2) that preceded the target number (i.e., participants responded by verbally producing the numbers). The training trials began with a center fixation for 500 ms, followed by a blank screen for 250 ms. Next, the flag cue was presented for 250 ms and the target number then appeared on the screen for participants’ responses (maximum duration: 2000 ms). The flag cues were presented in a pseudo-random order with a maximum of 3 consecutive trials of the same type of cue. There was an inter-stimulus interval of 500 ms before the onset of the next trial. The ratio of repetition and switch trials was 1:1 for each language in both blocks. Each training session lasted for approximately 15 min. Again, there was a total of eight training sessions during four consecutive days: two sessions per day, once in the morning and once in the afternoon. On each training day, the time interval between the two training sessions was identical to avoid the potential confounds of training mode.

#### Cognitive Control Measures: Color-Shape Switching and Anti-saccade Tasks

Participants in both groups received two tasks in the pre-training and post-training phases. The first task, the color-shape switching task, was a non-verbal experiment based on Prior and MacWhinney (2010) consisting of two single blocks (one pure color and one pure shape) and one mixed block. Each trial began with the presentation of a center fixation cross for 500 ms followed by a blank screen for 250 ms. The target item then appeared in the center of the screen until the participant responded (maximum duration: 4000 ms). The target set included a blue circle, a blue triangle, a red circle, and a red triangle. The inter-stimulus interval was 250 ms. Participants were instructed to place the left-hand middle and index fingers on the “Q” and “W” keys, respectively, for the color task; and the right-hand middle and index fingers on the “O” and “P” keys, respectively, for the shape task. In the pure color block, the participants were instructed to press the “W” key if the target was red and the “Q” key if the target was blue. In the pure shape block, they were asked to press the “P” key if the target was a triangle and the “O” key if the target was a circle. In the mixed block, a pre-cue preceded the target for 250 ms. Participants were instructed to make a color decision about the target if the pre-cue was a rainbow figure and a shape decision on the target if the pre-cue was a geometric figure. They were required to respond as quickly as possible based on the pre-cue dimension (color or shape). The blocks (two single blocks and one mixed block) were counterbalanced across participants. Each single block contained 8 practice trials, followed by 24 experimental trials that were randomly presented. The ratio of repetition and switch trials was 1:1 for each task and in both blocks. The mixed block included 16 practice trials (four for each kind of target) and 50 experimental trials. The shape and color tasks in the mixed blocks were randomly ordered with a maximum of four consecutive trials of the same type. Following Prior and MacWhinney, two additional dummy trials were added at the beginning of each block and were excluded in the analysis.

Our second task which measured cognitive control was the anti-saccade task. We modeled this experiment after Paap and Greenberg (2013) in terms of material, design, and procedure. The task included one anti-saccade block and one control block, counterbalanced across participants. The anti-saccade trials started with a center fixation, presented at various durations (600/1000/1400/1820/2200 ms), that was followed by a blank screen for 250 ms. A motionless symbolic distracter (i.e., “#”) then flickered about 2° to one side of fixation *twice* (each flicker duration was 100 ms with a blank screen of 50 ms in between). Upon the offset of the second flickering, the target letter (i.e., “B”, “P”, or “R”) was presented for 150 ms about 2° next to the fixation that was opposite to the distractor, followed by a visually similar mask (i.e., “8”) in the same location. Participants were instructed to identify the target letter by pressing the corresponding labels using three fingers of the right hand. Each letter was assigned different labels for the correct response. There was an inter-stimulus interval of 500 ms before the onset of the next trial. According to Paap and Greenberg, when the eventual target is presented on the opposite side of the distracter, the best strategy is to inhibit the reflexive, automatic urge to attend to a visual stimulus (i.e., distracter) that appears abruptly in the peripheral visual field. Thus, faster RTs should be expected if bilinguals are superior in their inhibitory control ability. For the control trials, the target letters appeared on the center of the screen after the center fixation with no presentation of the opposite field distracter.

The trials were organized as follows: The anti-saccade block consisted of 12 practice trials and 60 anti-saccade trials in which the presence of the target letters was counterbalanced between the left and right sides of the screen. In the control block, participants performed six practice trials, in which three target letters were presented two times each in a random order; and 30 control trials which were formed by the random combination of the aforementioned five fixation durations. Again, the sequence of the anti-saccade block and control block was counterbalanced across participants.

### Results

Only trials with correct responses were included in the analyses. RTs slower than 200 ms and greater than 2000 ms were excluded from the analyses. We also discarded trials whose RTs were 2.5 SDs below or above the mean (Liu et al., 2019a,b). This data-trimming procedure applied to both anti-saccade (12.6% excluded) and color-shape switching tasks (8.8% excluded). Considering the high accuracy of each task, our analyses focused mainly on RTs. We conducted multilevel mixed effects models with participants and trial order as crossed random effects in the R computing environment (lme4 package, Bates et al., 2007; lmerTest package, Kuznetsova et al., 2014). The reason for using mixed-effect models was to allow random effects of participants and trial order to be considered simultaneously (Baayen et al., 2008). Their results also are more reliable than traditional statistics (Barr et al., 2013).

For each task, we employed linear models for the RT data and included multiple fixed effects of theoretical interest (i.e., test, group, task condition, and their full interactions). All variables were contrast-coded, yielding tests of the main effects directly analogous to those obtained from an ANOVA (Fraundorf and Jaeger, 2016; Tullis and Fraundorf, 2017). As for random effects, we assessed the contribution of each random slope to each model by using likelihood-ratio tests and reported the best-fitting model with the maximal random effects structure justified by the data.

### Color-Shape Switching Task

In the data analyses for the color-shape switching task, one additional participant from the language switching training group was excluded due to low accuracy. This left 31 for the control group and 26 for the language switching training group to be included in the linear mixed effect model analysis. The mean RTs and mean accuracy are shown in Table 2. The mixing costs were defined as the difference between the non-switch (i.e., repetition) trials in the mixed block and the trials in the single block (Prior and MacWhinney, 2010). We fit a linear mixed effect model with the fixed effect of test (pre-training, post-training), group (control, language switching training), task condition (single block, mixed block), and their full interactions. For the random effect, the best-fitting model included a random intercept for subjects and trial order; by-subject random slopes for test, task condition, and their interaction; and by-trial order for test, the interaction between test and task condition, and the interaction between test and group. Other random slopes did not improve model fit (*p* > 0.1) and were thus omitted.

**TABLE 2:** Mean RTs (and SDs) and accuracy (and SDs) of the color-shape switching task in Experiment 1.

		**Pre-training**		**Post-training**	
		**Single**	**Mix**	**Single**	**Mix**
RT (ms)	Control group	418 ± 114	655 ± 237	425 ± 123	594 ± 194
	Language switching training group	479 ± 152	806 ± 349	383 ± 96	538 ± 176
ACC (%)	Control group	97 ± 18	94 ± 24	96 ± 19	97 ± 15
	Language switching training group	96 ± 18	94 ± 23	94 ± 23	94 ± 23

**FIGURE 1:**
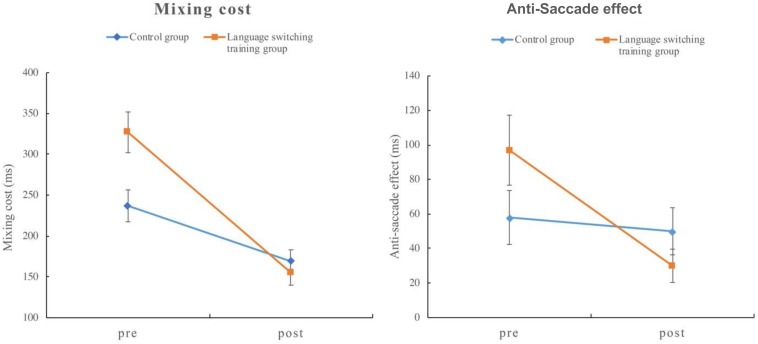
Mixing cost of the color-shape switching task **(left)** and anti-saccade effect of the anti-saccade task **(right)** in Experiment 1. Error bar represents standard error.

The results of the mean RTs are summarized in Table 3. These analyses showed a significant effect for test (*t* = 9.11, *p* < 0.001), suggesting overall faster responses in the post-training phase relative to the pre-training phase. There was also a significant effect for task condition (*t* = 16.08, *p* < 0.001), indicating slower responses of the repetition trials in the mixed block compared to the trials in the single block. In our design, the interactions relating to the task condition were the crucial index for the mixing costs, especially as demonstrated by the three-way interaction. The interactive effect between the test and the task condition was significant (*t* = 6.40, *p* < 0.001), indicating that mixing costs in the post-training were significantly smaller than those in the pre-training. Crucially the three-way interaction for test ^*^ group ^*^ task condition in the color-shape switching task was significant (*t* = −3.28, *p* < 0.001). Further analyses of this interaction suggest a greater reduction of the mixing costs from pre- to post-training for the language switching training group (172 ms) compared to the control group (68 ms) (see the left graph in Figure 1). Such improvement for the language switching training group suggests greater gains of monitoring ability from the switching-task training which we interpret as evidence for a training effect.

**TABLE 3:** Model parameters for the best-fitting linear mixed effects model of the color-shape switching task in Experiment 1.

**Model parameters**	**Estimate**	**SE**	***t***	***p***
(Intercept)	540.95	10.50	51.53	<0.001
Test	105.68	11.60	9.11	<0.001
Task condition	225.31	14.01	16.08	<0.001
Group	–30.85	17.85	–1.72	0.08
Test: Task condition	119.28	18.63	6.40	<0.001
Test: Group	–150.60	21.42	–7.03	<0.001
Group: Task condition	–36.95	20.68	–1.78	0.07
Test: Group: Task condition	–106.76	32.55	–3.28	<0.001
				

#### Anti-saccade Task

The descriptive results (i.e., mean RTs and mean accuracy) are reported in Table 4. The anti-saccade effect was assessed by subtracting the mean RT of the control block from that of the anti-saccade block (Paap and Greenberg, 2013). We fit a linear mixed effect model with the fixed effect of test (pre-training, post-training), group (control, language switching training), task condition (control, anti), and their full interactions. For the random effect, the best-fitting model included random intercept for subjects and trial order; by-subject random slopes for test and task condition; and by-trial order for task condition. Other random slopes did not significantly improve model fit and were omitted (*p* > 0.1).

**TABLE 4:** Mean RTs (and SDs) and mean accuracy (and SDs) of the anti-saccade task in Experiment 1.

		**Pre-training**		**Post-training**	
		**Control**	**Anti**	**Control**	**Anti**
RT (ms)	Control group	603 ± 153	661 ± 152	614 ± 158	664 ± 158
	Language switching training group	704 ± 193	801 ± 198	555 ± 131	585 ± 121
ACC (%)	Control group	93 ± 24	85 ± 35	95 ± 21	91 ± 28
	Language switching training group	91 ± 28	87 ± 33	93 ± 25	90 ± 30

The main effects of both test and task conditions were significant (*p* < 0.001) (see Table 5). The mean RT in the post-training phase was shorter than the mean RT in the pre-training phase, and the mean RT of the anti-saccade block was longer than the mean RT of the control block. We focused on the interactions that were related to the task condition because of our major theoretical motive in the anti-saccade effect. The interactive effect between test and task condition was significant (*t* = −7.17, *p* < 0.001), indicating that the anti-saccade effect in the post-training was significantly smaller than the anti-saccade effect in the pre-training. Same as the color-shape switching task, the three-way interaction for test ^*^ group ^*^ task condition was significant, demonstrating a difference between the control and language switching training groups in terms of the anti-saccade effect (*t* = 5.34, *p* < 0.001). As shown in Table 5, the language switching training group exhibited a greater decrease in the anti-saccade effect from pre- to post-training (67 ms) compared to the control group (8 ms). A visual representation of this finding can be seen in the graph on the right in Figure 1. Similar to the color-shape switching task, this greater improvement for the language switching training group suggests greater gains of inhibitory control ability from the switching-task training and supports evidence for a training effect.

**TABLE 5:** Model parameters for the best-fitting linear mixed effects model of the anti-saccade task in Experiment 1.

**Model parameters**	**Estimate**	**SE**	***t***	***p***
(Intercept)	648.94	10.43	62.18	<0.001
Test	89.17	12.65	7.04	<0.001
Task condition	–68.36	8.81	–7.75	<0.001
Group	–23.49	20.21	–1.16	0.25
Test: Task condition	–40.77	5.68	–7.17	<0.001
Test: Group	–190.16	25.30	–7.51	<0.001
Group: Task condition	8.08	16.15	0.50	0.61
Test: Group: Task condition	60.70	11.36	5.34	<0.001

### Discussion

The results of Experiment 1 show that language switching training reduced mixing costs and the anti-saccade effect within cognitive control in bilinguals, which suggest that a bilingual language switching experience could facilitate monitoring and inhibition. These results were consistent with the bilingual executive processing advantage hypothesis (Hilchey and Klein, 2011) and the inhibitory control model (Green, 1998), suggesting a positive relationship between BLC and EC. As mentioned above, during language switching, bilinguals need to monitor the communicative environment for changes that trigger a language switch and subsequently inhibit the non-target language when speaking in the intended language. For the language switching training group, monitoring and inhibitory control ability were enhanced through intensive language switching training compared to the control group.

While the findings of Experiment 1 show that the language switching training facilitated monitoring and inhibitory control within cognitive control, they do not tell us specifically *where* this facilitative effect comes from. This is due to the fact that we could not separate the monitoring processing and inhibitory control processing during one language switching task. To address this, in Experiment 2, one group of participants received a series of L2 training which only engaged inhibition processing (L2 training group) while another group received language switching training as in Experiment 1. According to the adaptive control hypothesis (Green and Abutalebi, 2013), certain components of cognitive control show facilitative effects when the context imposes demands on it. Hence, the training of monitoring during language switching would facilitate monitoring ability within cognitive control and the training of inhibitory control during language switching would facilitate inhibitory control ability within cognitive control. If this is the case, then a greater decrease of mixing cost and a smaller decrease of anti-saccade effect from pre- to post-training for the language switching training group compared to the L2 training group should be observed.

## Experiment 2

### Participants

Fifty Chinese-English bilinguals (all non-English majors) who did not participate in Experiment 1 but were from the same population, participated in Experiment 2. The ages ranged from 18 to 24 (*M* = 20.2, *SD* = 1.6). These individuals were all right-handed with normal or corrected-to-normal vision. As in Experiment 1, all participants were EFLs, with a mean age of first exposure to English of 8 years (*SD* = 2.9) and were also asked to complete a language background questionnaire. The two groups were not significantly different in their language background (*p* > 0.1). Table 6 shows participants’ language background information, including L2 AoA, L2 proficiency, and frequency of language switching. One participant was excluded from data analysis due to test incompletion. The remaining participants were randomly and evenly divided into two groups: 24 for the language switching training group and 25 for the L2 training group. All participants provided written informed consent before the experiment and were informed of their right to withdraw at any time. Each participant received monetary compensation for participation.

**TABLE 6:** Language background information for participants in Experiment 2.

	**L2 AoA**	**L2 proficiency**	Frequency of language switching^*^
L2 training group	8.4 ± 2.1	4.0 ± 1.0	2.7 ± 0.5
Switching training group	8.3 ± 2.7	4.2 ± 0.8	2.8 ± 0.4

^*^ As measured by a language switching questionnaire (Rodriguez-Fornells et al., 2012).

### Design and Procedure

As in Experiment 1, Experiment 2 was conducted in three phases (i.e., a pre-training phase, a training phase and a post-training phase). Participants of both language switching training and L2 training groups were asked to complete the same non-linguistic cognitive control tasks (the anti-saccade task and the color-shape switching task) in both pre- and post-training phases. During the training phase, participants in both groups were required to complete the training task for a total of four times (instead of eight times in Experiment 1) on four consecutive days. However, each training time in Experiment 2 lasted for approximately 30 min rather than 15 min as in Experiment 1. We reduced the number of training days but maintained the same amount of time to avoid participant attrition due to multiple sessions. Participants in the language switching training group were instructed the same as those in Experiment 1 to perform a cued naming task which required high demands of switching between languages whereas participants in the L2 training group were instructed to name all stimuli only in L2, and thus they did not switch between languages. All other aspects of the design and procedure were the same as those in Experiment 1.

### Results

The procedure of data trimming and analysis was the same as in Experiment 1. Only trials with correct responses were included in the analyses.

#### Color-Shape Switching Task

**FIGURE 2:**
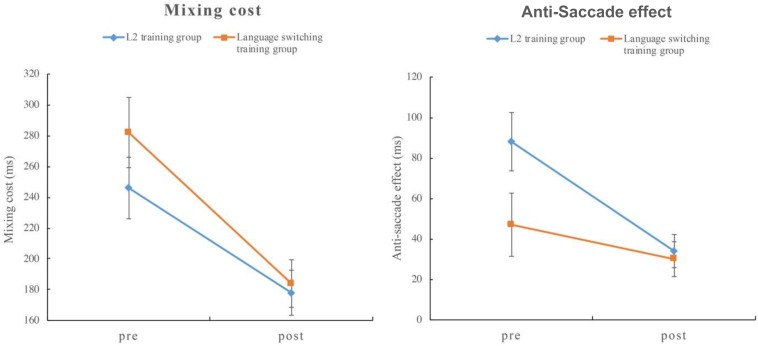
Mixing costs of the color-shape switching task **(left)** and anti-saccade effect of the anti-saccade task **(right)** in Experiment 2. The error bar represents standard error.

Data from both groups were entered into a linear mixed effect model analysis. Applying the same data trimming procedure as above resulted in 8.6% data exclusion. Table 7 shows the mean RTs and accuracy and Table 8 summarizes the results of the mean RTs. Like Experiment 1, both the test and the task conditions were significant (*p* < 0.001), indicating that participants responded faster in the post-training phase than in the pre-training phase and slower for the repetition trials in the mixed block compared to the trials in the single block. The interactive effect between the test and the task condition was significant (*t* = 11.26, *p* < 0.001), indicating that the mixing costs in the post-training were significantly smaller than those in the pre-training. More importantly, the three-way interaction for test ^*^ group ^*^ task condition was significant (*t* = −2.18, *p* = 0.02), suggesting that the language switching training group showed a greater decrease in mixing costs from pre- to post-training (98 ms) compared to the L2 training group (68 ms) (see left graph in Figure 2). This pattern is consistent with that of Experiment 1 in that both the control group in Experiment 1 and the L2 training group in Experiment 2 do not involve monitoring process.

**TABLE 7:** Mean RTs (and SDs) and mean accuracy (and SDs) of the color-shape switching task in Experiment 2.

		**Pre-training**		**Post-training**	
		**Single**	**Mix**	**Single**	**Mix**
RT (ms)	L2 training group	437 ± 114	683 ± 241	439 ± 126	617 ± 204
	Language switching training group	442 ± 110	724 ± 267	428 ± 106	612 ± 214
ACC (%)	L2 training group	94 ± 23	92 ± 27	96 ± 19	94 ± 22
	Language switching training group	97 ± 18	94 ± 24	96 ± 19	96 ± 20

**TABLE 8:** Model parameters for the best-fitting linear mixed effects model of the color-shape switching task in Experiment 2.

**Model parameters**	**Estimate**	**SE**	***t***	***p***
(Intercept)	552.26	11.29	48.89	<0.001
Test	48.70	7.92	6.14	<0.001
Task condition	226.18	15.55	14.53	<0.001
Group	–8.60	19.85	–0.43	0.66
Test: Task condition	85.52	7.59	11.26	<0.001
Test: Group	–36.82	15.84	–2.32	0.02
Group: Task condition	–24.10	22.02	–1.09	0.27
Test: Group: Task condition	–33.19	15.18	–2.18	0.02

#### Anti-saccade Task

Data from one participant in the L2 training group was excluded due to low accuracy, resulting in 24 participants in the L2 training group and 24 in switching training group. The same data-trimming criteria described above resulted in 10.6% data exclusion. Table 9 shows the mean RTs and mean accuracy for the two groups in the anti-saccade task and Table 10 summarizes the results of the mean RTs. Following the same analysis procedure of fitting a linear mixed effect model, both test and task conditions were again significant. Participants responded faster in the post-training phase than in the pre-training phase, and they responded slower in the anti-saccade block than in the control block. Both the interaction for test ^*^ task condition (*t* = −6.75, *p* < 0.001) and, crucially, the three-way interaction for test ^*^ group ^*^ task condition (*t* = −2.56, *p* = 0.01) were significant. Resolution of the interaction revealed that, consistent with our hypothesis, it is now the L2 training group that showed a greater decrease of the anti-saccade effect from pre- to post-training (54 ms) compared to the language switching training group (17 ms) (see right graph in Figure 2). These results arise from more demands on inhibitory control for the L2 training group than for the language switching training group.

**TABLE 9:** Mean RTs (and SDs) and mean accuracy (and SDs) of the anti-saccade task in Experiment 2.

		**Pre-training**		**Post-training**	
		**control**	**anti**	**control**	**anti**
RT (ms)	L2 training group	631 ± 145	719 ± 150	618 ± 140	652 ± 126
	Language switching training group	682 ± 192	729 ± 162	642 ± 168	672 ± 150
ACC (%)	L2 training group	93 ± 26	89 ± 31	95 ± 21	92 ± 27
	Language switching training group	93 ± 24	90 ± 29	94 ± 23	93 ± 24
					

**TABLE 10:** Model parameters for the best-fitting linear mixed effects model of the anti-saccade task in Experiment 2.

**Model parameters**	**Estimate**	**SE**	***t***	***p***
(Intercept)	669.81	12.63	52.99	<0.001
Test	41.23	7.50	5.49	<0.001
Task condition	–52.21	7.32	–7.12	<0.001
Group	–26.20	24.86	–1.05	0.29
Test: Task condition	–41.33	6.12	–6.75	<0.001
Test: Group	–5.62	14.69	–0.38	0.70
Group: Task condition	–22.54	13.50	–1.66	0.11
Test: Group: Task condition	–30.74	12.00	–2.56	0.01

### Discussion

The findings of Experiment 2 showed a greater decrease in mixing costs and a smaller decrease in the anti-saccade effect from pre- to post-training for the language switching training group compared to the L2 training group. Combined with the results in Experiment 1, both suggest a bilingual facilitative effect on monitoring and inhibitory control. These findings are consistent with the bilingual executive processing advantage hypothesis (Hilchey and Klein, 2011) and the inhibitory control model (Green, 1998).

Critically, the results in Experiment 2 were able to illuminate the finding that the bilingual facilitative effect seems to emerge from training on both monitoring and inhibitory control during language switching. Training monitoring during language switching facilitates monitoring abilities within cognitive control and training inhibitory control facilitates inhibitory control abilities within cognitive control. These findings can be explained by the adaptive control hypothesis (Green and Abutalebi, 2013) which proposes that the control processes themselves can adapt to the demands imposed on them by context. Specifically, in the present study, different training contexts placed different demands on the control processes under scrutiny (i.e., monitoring or inhibitory control), which eventually improve the specific monitoring or inhibitory control abilities.

## General Discussion

Our empirical focus is the debate on whether BLC overlaps two componential processes of EC: monitoring and inhibitory control. Instead of using a traditional cross-sectional approach the current study adopted a training design in which participants received language switching training and completed the same cognitive control tasks in the pre- and post-training. By comparing the performance of a language switching training group and a control group from pre- and post-training in Experiment 1, we found that language switching training significantly enhanced the performance of monitoring and inhibitory control within EC, to some extent, suggesting a positive relationship between BLC and EC. Such relationship was demonstrated by the gains of the inhibitory control component (i.e., anti-saccade effect) in the anti-saccade task, and the monitoring component (i.e., mixing costs) in the color-shape switching task. Furthermore, by comparing the performance of the language switching training group and the L2 training group from pre- and post-training in Experiment 2, we found that the observed positive relationship between BLC and two components process of EC (i.e., monitoring and inhibitory control) arise from the corresponding training on both monitoring and inhibitory control during language switching.

### The Positive Relationship Between BLC and EC

Regarding the ongoing debate as to whether BLC overlaps with EC or not, previous researchers mainly have conducted studies with cross-sectional designs to examine facilitative effects on EC among bilinguals. In contrast, by using a training design, the current study uniquely examined two components of EC based on the two main control processing in language switching, namely monitoring and inhibitory control. Because the monitoring and inhibitory control processes contained in language switching received intensive exercises during the training phase, such abilities improved and further facilitated the performance of corresponding domains of EC.

Mixing costs in switching tasks are typically used to assess the processing of monitoring components (Braver et al., 2003; Prior and MacWhinney, 2010). Our results in Experiment 1 showed evidence for a positive relationship between BLC and monitoring such that language switching training led to significant reductions in mixing costs, suggesting that the adaptive changes resulted from our language switching training mainly reflected monitoring processing. The facilitative effect on monitoring processes has been demonstrated by previous studies. Bialystok et al. (2005) reported reduced mixing costs for bilinguals by comparing mixed blocks with single-task blocks. Costa et al. (2009), for example, using a flanker task, found an advantage for bilinguals, relative to monolinguals, on conflict monitoring processing under demanding circumstances (i.e., 50% or 75% congruent trials, at least 25% intermixed incongruent trials). However, the bilingual effects on monitoring components (i.e., mixing costs) are still in dispute. For example, two recent studies adopting similar non-linguistic switching tasks provide a comparison to our results. Prior and MacWhinney reported that there was no bilingual advantage compared to monolinguals in terms of mixing costs. In contrast, Soveri et al. (2011) found that bilinguals outperformed monolinguals in the mixing costs. One possible reason for this may be due to confounding factors contained in traditional cross-sectional designs. Instead, by using the training method, we found a facilitative effect on mixing costs, supporting the bilingual advantage in monitoring ability (Hilchey and Klein, 2011), which is consistent with the findings from a recent study by Timmer et al. (2019).

Inhibitory control has been recognized as a core element of EC (Diamond, 2013). Beyond the facilitative effect in monitoring, the findings in Experiment 1 also showed that specific training on inhibition during language switching facilitates the inhibition process in EC, suggesting a positive relationship between BLC and inhibitory control. This result is consistent with previous studies demonstrating that bilingualism exerts positive effects on inhibitory control processes (Bialystok and Viswanathan, 2009). However, Paap and Greenberg (2013), with similar employment of the anti-saccade task, reported no cognitive advantage of inhibitory control processing for bilinguals compared to monolinguals. Given that our study differed from Paap and Greenberg—primarily in that ours is based on a training design whereas Paap and Greenberg’s used cross-sectional comparisons between monolinguals and bilinguals—it seems likely that these differences might contribute to the inconsistencies found in research investigating these issues.

Taken together, the current study indicates that short-term bilingual language switching training facilitates inhibitory control and monitoring processing within EC, which is in line with bilingual executive processing advantage hypothesis (Hilchey and Klein, 2011) and inhibitory control model (Green, 1998), supporting the bilingual advantage effect on monitoring and inhibitory control. These results further suggested a positive relationship between BLC and EC. However, we note that these positive relationships are observed in a short-term, experimental setting which is different from the cross-sectional studies. The latter have examined whether bilinguals compared to monolinguals show improved cognitive control due to the long-term, daily practice with language switching that bilinguals have. As daily language switching is not as intense as was our language switching training protocol in the current study, we could conclude that there are positive effects of short-term language switching training on inhibitory control or monitoring abilities. However, we cannot disentangle whether long-term daily language switching experience could facilitate such cognitive control abilities (for a review, see Blanco-Elorrieta and Pylkkänen, 2016).

### The Sources of the Positive Relationship Between BLC and EC

Most of the studies that have reported a positive relationship between BLC and EC have only focused on whether such a relationship existed or not, without further investigating the source of the positive relationship they observed (Bialystok et al., 2006; Dong and Xie, 2014; Liu et al., 2016; among others). In Experiment 2, we found the L2 training group showed a greater decrease in the anti-saccade effect from pre- to post-training (54 ms) compared to the language switching training group (17 ms). This is because the L2 training group had more demand for inhibitory control than the language switching training group. For the L2 training group in our study, mainly inhibitory control is engaged. Moreover, the participants in the present study are unbalanced Chinese-English bilinguals who lived in the dominant L1 (i.e., Chinese) environment with very low proficiency in their L2 language (i.e., English). According to inhibitory control model (Green, 1998), since L1 initially has a larger activation than L2, more inhibition will be needed to suppress L1 during L2 production than vice versa. Specifically, naming in L2 requires inhibiting the competing response in the L1, as well as the task goal of speaking in the L1, and unbalanced bilinguals must rely on strong inhibition of the L1 in order for production in the L2. Conversely, when producing in a highly dominant L1, unbalanced bilinguals need to inhibit the L2 to a lesser degree (Costa and Santesteban, 2004; for a review, see Declerck and Philipp, 2015). By contrast, for the language switching group, both monitoring and inhibitory control are engaged, and more importantly, the inhibitory control contains both strong inhibition of L1 and weak inhibition of L2. Thus, the overall inhibition for the language switching group is smaller than the L2 training group. On the other hand, the language switching training group showed a greater decrease of mixing costs from pre- to post-training (98 ms) compared to the L2 training group (68 ms), due to the fact that the language switching training group demanded more monitoring than the L2 training group as there is little conflict monitor processing during L2 training with only one language. These results together indicated that the bilingual facilitative effects on monitoring and inhibitory control derived from the corresponding specific training of these abilities during language switching, which provided empirical evidence for the source of the positive relationship between BLC and EC.

Also in Experiment 2, one group of participants received a series of language switching training sessions that engaged monitor and inhibitory control processes (language switching training group), whereas the other group received L2 training that only engaged inhibition processing (L2 training group). The results showed a greater decrease of mixing cost from pre- to post-training for the language switching training group compared to the L2 training group, and a greater decrease of the anti-saccade effect from pre- to post-training for the L2 training group compared to the language switching training group, suggesting that the observed positive relationship between BLC and EC derived from the training on both monitoring and inhibition during language switching. Specifically, the short-term training on monitoring during language switching in an experimental setting facilitates the monitoring process within cognitive control and the training on inhibitory control during language switching facilitates the inhibitory control process within cognitive control.

As mentioned above, these findings are in line with the adaptive control hypothesis (Green and Abutalebi, 2013), which proposes that depending on the context, language control processes may change the way they work or the way they cooperate with other processes. The findings in the present study show the after-effects adapting to the contexts. Specifically, the bilingual facilitative effect in monitoring and inhibitory control reflected the adaptive changes to the demand imposed by the language switching training contexts. During the training sessions, participants needed to name the target number in accordance with the flag cue. To do so, participants had to choose the accurate language by continuously monitoring the flag cue, then inhibit the interference from the non-target language using inhibitory control processes. Such intense training on monitoring and inhibition during language switching in an experimental setting led to facilitative effects on corresponding domains of EC.

In sum, combined with the observed positive relationship between BLC and the two components of EC (i.e., monitoring and inhibitory control) in Experiment 1, and the empirical evidence for the sources of such positive relationship in Experiment 2, it is reasonable to believe that the observed positive relationship between BLC and EC is reliable, at least in an experimental setting.

### Limitations

One limitation of the current study is the lack of training the control group received in the first experiment. These leaves open whether the observed training effects are related to training in general or to the specific language training that the experimental group underwent. Particularly, it is unclear as to whether it is the “switching” aspect or the “language” aspect of the training that is driving the effects, as other studies have shown that other more general task-switching training also improves monitoring and inhibitory control (e.g., Karbach and Kray, 2009). More research needs to be conducted to examine these issues further. Finally, another limitation is that the experimental groups were not well matched at pre-training, although we divided the participants randomly. This limitation may bring some confounding effects as these participants may have been closer to ceiling performance and thus, may not have had that much room to improve.

## Conclusion

The present study reveals that there is a positive relationship between BLC and two components of EC: monitoring and inhibitory control, and such positive relationship can be observed as a result of short-term training on both monitoring and inhibitory control during language switching in an experimental setting. These findings not only inform the ongoing debate on the relationship between BLC and EC, but they also open the door for discussion as to how these issues can be explained through other theoretical lenses. Future studies are encouraged and merited that will help explore the potential cognitive benefits of bilingualism through complementary lines of thought.

## Data Availability

The datasets generated for this study are available on request to the corresponding author.

## Ethics Statement

This study was carried out in accordance with the recommendations of the Human Research Ethics Committee for Non-Clinical Faculties at the School of Psychology of South China Normal University with written informed consent from all subjects. All subjects gave written informed consent in accordance with the Declaration of Helsinki. The protocol was approved by the Human Research Ethics Committee for Non-Clinical Faculties at the School of Psychology of South China Normal University.

## Author Contributions

CL, LJ, and RW designed the study. CL, LJ, and XS acquired and analyzed the data. CL, LJ, CY, JS, and RW wrote the manuscript.

## Conflict of Interest Statement

The authors declare that the research was conducted in the absence of any commercial or financial relationships that could be construed as a potential conflict of interest.

## Footnotes

1 Although today, Hilchey and Klein (2011) no longer support their bilingual executive processing advantage hypothesis.
